# Carbapenemase-Producing Enterobacteriaceae Isolates from Edo State, Nigeria

**DOI:** 10.1128/AAC.00255-17

**Published:** 2017-07-25

**Authors:** Christiana Jesumirhewe, Burkhard Springer, Sarah Lepuschitz, Franz Allerberger, Werner Ruppitsch

**Affiliations:** a Department of Pharmaceutical Microbiology, Prof. Dora Akunyili College of Pharmacy, Igbinedion University, Okada, Edo State, Nigeria; b Institute of Medical Microbiology and Hygiene, Austrian Agency for Health and Food Safety (AGES), Vienna, Austria

**Keywords:** carbapenem-resistant Enterobacteriaceae

## LETTER

The emergence and spread of carbapenem-resistant Enterobacteriaceae (CRE) are a global health problem that is of great concern to public health services (1, 2). The purpose of this study was to determine the frequency of CRE in three Nigerian hospitals and to characterize the resistance mechanisms of such isolates.

A total of 218 consecutive clinical isolates of Enterobacteriaceae based on inclusion criteria were collected from March to May 2015 at three medical microbiology laboratories of hospitals in Edo State, Nigeria (see Table S1 in the supplemental material). Screening for carbapenem resistance was performed using meropenem and ertapenem discs (10 μg; Oxoid, United Kingdom) according to EUCAST guidelines (3). The Kirby-Bauer susceptibility testing technique (4) and Etest method were carried out, and results were interpreted using EUCAST criteria (5). Identification of the involved resistance mechanisms was determined by whole-genome sequencing (WGS).

Out of 218 consecutive clinical Enterobacteriaceae isolates, 9 (4.1%) were further investigated due to cutoff values above the EUCAST screening recommendations (Table 1). All isolates showed resistance to piperacillin-tazobactam and amoxicillin-clavulanic acid, all but isolate Ec4349 showed resistance to fluoroquinolones and cefotaxime, and all but two isolates each showed resistance to ceftazidime (Ec4349 and Ecl2840_1) and ertapenem (Ec4349 and Ecl10_14_15). Only Ec4349, Ecl2845, Ecl2840_1, and EclQ9 were sensitive to cefepime and aztreonam. The carbapenemase inactivation method (6) revealed positive results for all nine CRE isolates. By application of WGS, one Klebsiella pneumoniae isolate harbored the *bla*_OXA-181_ gene; two K. pneumoniae isolates harbored the *bla*_NDM-1_ gene. The *bla*_OXA-48_ gene was detected in one Escherichia coli isolate, one K. pneumoniae isolate, and four Enterobacter cloacae isolates, respectively. All isolates had a single carbapenemase resistance gene on their draft genome fragment. Thirteen plasmid incompatibility groups were identified among the nine CRE isolates. Multilocus sequence typing (MLST) grouped the nine isolates into five sequence types.

**TABLE 1:** Characteristics of carbapenem-resistant Enterobacteriaceae isolates

Isolate^*a*^	Hospital^*b*^	Clinical specimen	MIC values (μg/ml)^*c*^	Resistance genes	ST^*d*^	GenBank accession no.^*e*^	Plasmid replicon(s)^*f*^
Kp1337LF	UBTH	Urine	MEM, 0.75; ETP, 3; COL, 1	*aac(6')lb-cr*, *aac(3)-lla*, *str A*, *aadA1*, *str B*, *bla*_TEM-1B_, *bla*_CTX-M-15_, *bla*_SHV-11_, *bla*_OXA-181_, *bla*_OXA-1,_ *mph*(A), *catA1*, *catB3*, *qnrS1*, *qnrB2*, *oqxA*, *oqxB*, *sul1*, *tet*(A), *dfrA15*	11	SAMN06704546	IncFIB(Mar), ColKP3, IncX3,* IncFII(K), IncFIB(K), IncR
Kp852	UBTH	Urine	MEM, 24; ETP, 32; COL, 1	*aadA1*, *aac(3)-lla*, *aacA4*, *aph(3')-Vla*, *arm A*, *aadA2*, *aac(6')lb-cr*, *bla*_SHV-28_, *bla*_CTX-M-15_, *bla*_NDM-1_, *bla*_OXA-1_, *msr*(E), *mphE*, *catB3*, *oqxB*, *oqxA*, *aac(6')-lb-cr*, *qnrB1*, *sul1*, *tet*(A), *dfrA12*	15	SAMN06704523	Col(BS512), IncFIB(pKPHS1), IncFII, IncFIA(HI1), IncR, IncFIB(K), IncFIB(Mar), IncHI1B*
Kp852K	UBTH	Urine	MEM, 32; ETP, 24; COL, 0.75	*aadA1*, *aac(3)-lla*, *aacA4*, *aph(3')-Vla*, *arm A*, *aadA2*, *aac(6')lb-cr*, *bla*_SHV-28_, *bla*_CTX-M-15_, *bla*_NDM-1_, *bla*_OXA-1_, *msr*(E), *mphE*, *catB3*, *oqxB*, *oqxA*, *aac(6')-lb-cr*, *qnrB1*, *sul1*, *tet*(A), *dfrA12*	15	SAMN06704522	Col(BS512), IncFIB(pKPHS1), IncFII, IncFIA(HI1), IncR, IncFIB(K), IncFIB(Mar), IncHI1B*
Kp872	UBTH	Endocervical swab	MEM, 4; ETP, 1.5; COL, 0.38	*str A*, *str B*, *aadA2*, *aac(3)-lla*, *bla*_CTX-M-15_, *bla*_OXA-48_, *bla*_TEM-1B_, *bla*_SHV-11,_ *oqxB*, *oqxA*, *sul1*, *sul2*, *tet*(D), *dfrA14*	340	SAMN06704513	IncFIA(HI1), IncR, IncL/M(pOXA-48)*
Ecl10_14_15	CH	Peritoneal fluid	MEM, 1.5; ETP, 0.75; COL, 0.75	*bla*_ACT-5_, *bla*_OXA-48_, *qnrB1*, *dfrA14*	78	SAMN06704512	IncL/M(pOXA-48)*
Ecl2845	UBTH	Urine	MEM, 0.75; ETP, 2; COL, 0.75	*bla*_ACT-5_, *bla*_OXA-48_	78	SAMN06704511	IncL/M(pOXA-48)*
EclQ9	UBTH	Unidentified source	MEM, 0.75; ETP, 1.5; COL, 0.75	*bla*_ACT-5_, *bla*_OXA-48_	78	SAMN06703827	IncL/M(pOXA-48)*
Ecl2840_1	UBTH	Urine	MEM, 4; ETP, 4; COL, 1	*bla*_ACT-5_, *bla*_OXA-48_	78	SAMN06703828	IncL/M(pOXA-48)*
Ec4349	UBTH	Urine	MEM, 0.25; ETP, 0.75; COL, 0.38	*str A*, *str B*, *bla*_TEM-1B_, *bla*_OXA-48_, *sul2*, *tet*(A), *dfrA14*	1408	SAMN06703826	Col3M, IncL/M(pOXA-48),* IncR

a Ec, *Escherichia coli*; Kp, *Klebsiella pneumoniae*; Ecl, Enterobacter cloacae.

b UBTH, University of Benin Teaching Hospital; CH, Central Hospital, Benin.

c MEM, meropenem; ETP, ertapenem; COL, colistin.

d ST, sequence type.

e Accession numbers are shown for the carbapenem resistance gene.

f *, plasmid replicon type harboring the carbapenem resistance gene.

Previous reports from Nigeria on molecular characterization of carbapenem resistance genes have identified genes such as *bla*_VIM_, *bla*_GES_, *bla*_NDM_, *bla*_OXA-181_, and *bla*_KPC_ (7–10). *bla*_OXA-48_, obtained from our study, has only been determined phenotypically. To the best of our knowledge, our findings, where six out of nine carbapenemase-producing isolates harbored the *bla*_OXA-48_ gene, represent the first molecular determination of the *bla*_OXA-48_ gene for Nigeria. The presence of different plasmid replicon types in carbapenemase-producing Enterobacteriaceae (CPE) underlines their importance in the dissemination of resistance genes. The IncL/M plasmid type was found in all of our OXA-48-producing isolates, correlating with previous reports indicating that the current spread of OXA-48 β-lactamase producers is mainly related to the diffusion of this plasmid (11).

Occurrence of CPE has been reported globally (12–14). In Nigeria, most previous reports characterized CRE phenotypically (15–17). Only a few studies used molecular methods, which are considered the “gold standard” for identification of carbapenemase-producing bacteria (12, 18). Carbapenem resistance is of particular concern as carbapenems are often the last available treatment option for infections due to multidrug-resistant Enterobacteriaceae (19). To the best of our knowledge, we are reporting the first genomic characterization of CRE from Nigeria. Detailed characterization of CRE is required to combat this worldwide emerging threat and improve patient outcomes.

## Accession number(s).

WGS results from our isolates have been deposited in GenBank under the accession numbers listed in Table 1.

## Supplementary Material

Supplemental material
