# Supplementary material for: Carbapenemase-Producing Enterobacteriaceae Isolates from Edo State, Nigeria
Source: Antimicrob Agents Chemother. 2017 Jul 25;61(8):e00255-17. doi: 10.1128/AAC.00255-17 (PMC5527642; doi:10.1128/AAC.00255-17)
Supplement: Supplemental material [file supp_61_8_e00255-17__index.html]

Supplemental material 

# Carbapenemase-Producing Enterobacteriaceae Isolates from Edo State, Nigeria

## Supplemental material

- Supplemental file 1 -

  Supplemental Table S1

  XLSX, 12K
